# Supplementary material for: Privacy in Community Pharmacies in Saudi Arabia: A Cross-Sectional Study
Source: Healthcare (Basel). 2024 Aug 31;12(17):1740. doi: 10.3390/healthcare12171740 (PMC11394820; doi:10.3390/healthcare12171740)
Supplement: Supplementary file 1 [file healthcare-12-01740-s001.zip › healthcare-3170692-supplementary.pdf]

File S1: Questionnaire

Privacy Questions	strongly agree	agree	Neutral	disagree	strongly disagree
There is a private space for me to talk to the pharmacist about some problems/medical questions.					
The design of community pharmacies takes into account patient privacy.					
Pharmacy customers give enough personal space between themselves and other customers when talking to the pharmacist.					
When I talk to the pharmacist about my health problems, I feel like I'm in a place that respects my privacy.					
Pharmacy customers can easily hear the conversations of other customers with the pharmacist.					
Do you feel that the pharmacist acts professionally when discussing your health information?					
Do you feel that the pharmacy provides you with a suitable environment to talk about your private health information?					
Do you think pharmacies take enough steps to educate customers about the importance of respecting the privacy of patients' health information?					
**Privacy Questions**	**5**	**4**	**3**	**2**	**1**
How important is it to have a private space for yourself when you are in a community pharmacy? Where 5 means very important and 1 means not important at all					
How do you rate your privacy concerns when you are in a community pharmacy? Where 5 means very concerned and 1 means not concerned at all					
**Privacy Questions**	**No**			**Yes**	
Have you ever been asked for unnecessary personal information when buying medication?					
Have you ever avoided discussing a health problem with the pharmacist due to privacy concerns?					
Do you believe that your personal health information is treated confidentially in pharmacies?					
Do you have any previous experience that indicates a privacy breach during your visit to a community pharmacy in Saudi Arabia?					
